# A novel targeted angiogenesis technique using VEGF conjugated magnetic nanoparticles and in-vitro endothelial barrier crossing

**DOI:** 10.1186/s12872-017-0643-x

**Published:** 2017-07-28

**Authors:** Mark Christopher Arokiaraj

**Affiliations:** Pondicherry Institute of Medical Sciences, Pondicherry, 605014 India

**Keywords:** Angiogenesis, Magnetic Nanoparticles, Tissue culture

## Abstract

**Background:**

Vascular endothelial growth factor is well known for its angiogenesis potential. The study was performed to determine the possible pro-angiogenic role of magnetic nanoparticles coupled to VEGF in vitro and their capacity to cross an endothelial monolayer. This novel treatment technique for angiogenesis could be potentially useful for therapeutic purposes using magnetic nanoparticles.

**Methods:**

Magnetic nanoparticles (MN) were synthesized and were conjugated with the vascular endothelial growth factor. The particles were tested in vitro in a 2D to 3D culture system. MN was seeded in different positions in relation to an HUVEC spheroid to assess a preferential migration.

To evaluate the MN capacity to cross the endothelial barrier, a confluent monolayer of HUVEC cells was seeded on top of a collagen gel. MN was placed in dissolution on the cell culture media, and the MN position was determined by confocal microscopy for 24 h.

**Results:**

HUVEC spheroids were able to generate a preferential sprouting depending on the MN position. Meanwhile, there was random migration when the MN’s were placed all over the collagen gel and no sprouting when no MN was added. The trans-endothelial migration capacity of the MN was observed after 20 h in culture in the absence of external stimuli.

**Conclusion:**

Here we show in vitro angiogenesis following the distribution of the MN conjugated with growth factors. These nanoparticles could be controlled with a magnet to place them in the ischemic area of interest and speed up vascular recovery. Also, MN has potentials to cross endothelium, opening the doors to a possible intravascular and extravascular treatment.

**Electronic supplementary material:**

The online version of this article (doi:10.1186/s12872-017-0643-x) contains supplementary material, which is available to authorized users.

## Background

Angiogenesis is a process wherein new vessels form in response to an ischemic or hypoxic stimuli [1, 2]. Angiogenesis is mediated through vascular endothelial growth factors, hypoxic ischemic growth factors, angiopoietic hormones, platelet derived growth factors and fibroblastic growth factors. Among all these factors VEGF plays a major role, and it exerts its effect not only by stimulation following hypoxic stimulus but also independently [3–6]. VEGF primarily acts by phosphatidylinositol 3-kinase pathway through hypoxia inducible factor-1 transcriptional element [7]. The promoter region of VEGF is heavily influenced by hypoxic-ischemic growth factors [8]. Coronary collaterals are angiogenesis observed in response to ischemia, and it is usually a slow process [9]. In patients where coronary interventions or bypass surgery are not feasible, the growth of therapeutic collaterals would be very useful to reduce ischemic symptoms [10, 11]. Moreover, these patients are often debilitated by the ischemic symptoms. Therefore, there is a definite need for a novel therapeutic method for coronary ischemia other than angioplasty and coronary arterial bypass grafting. Hence, a method of targeted angiogenesis in the ischemic areas would be very useful as a novel and challenging therapeutic measure [11]. In the past angiogenic gene injection has shown some effects on the collateral formation with minimal benefits. Invasive angiogenic protein growth factor treatment with basic fibroblast growth factor (bFGF) or VEGF was ineffective in placebo-controlled clinical trials [12, 13]. As direct injection of proteins is ineffective, in this study, we focused on a novel therapeutic development using certain biocompatible magnetic nanoparticles as a novel carrier with vascular endothelial growth factors for growth of coronary collaterals. There is also an age-dependent impairment of angiogenesis [14]. Targeted angiogenesis is a therapeutic challenge, which is essentially useful to overcome ischemia in a focused and less invasive method. Controlled growth of collaterals in required regions or ischemic areas would be very useful in treatment strategies. The magnetic control of the particles would help to navigate or retain the particles in required ischemic regions, as isolated growth factors alone cannot be controlled.

## Methods

Commercially available magnetic nanoparticles were acquired from NVIGEN Inc. USA with streptavidin on surface. Biotinylated vascular endothelial growth factor (Fluorokine) was acquired from MD systems Inc. USA. Thereafter, nanoparticles and growth factor conjugation was performed by standard techniques [15]. The size of the nanoparticles is in the range of 200 nm. To control the magnetic nanoparticles the required magnetic field gradient strength is approximately 10 T/M. Fluorescent tagging of the particles was performed using fluorescent conjugation. After completion of conjugation, the extent of release of the VEGF was studied. When the release of VEGF was confirmed the particles were taken up for tissue culture study. For setting up the experiment, standard techniques were followed [16, 17]. The experiments were setup in a vertical sandwich technique inside microfluidic chips. The tissue culture experiment was performed in a background of 5% CO_2_.

**Fig. 1:**
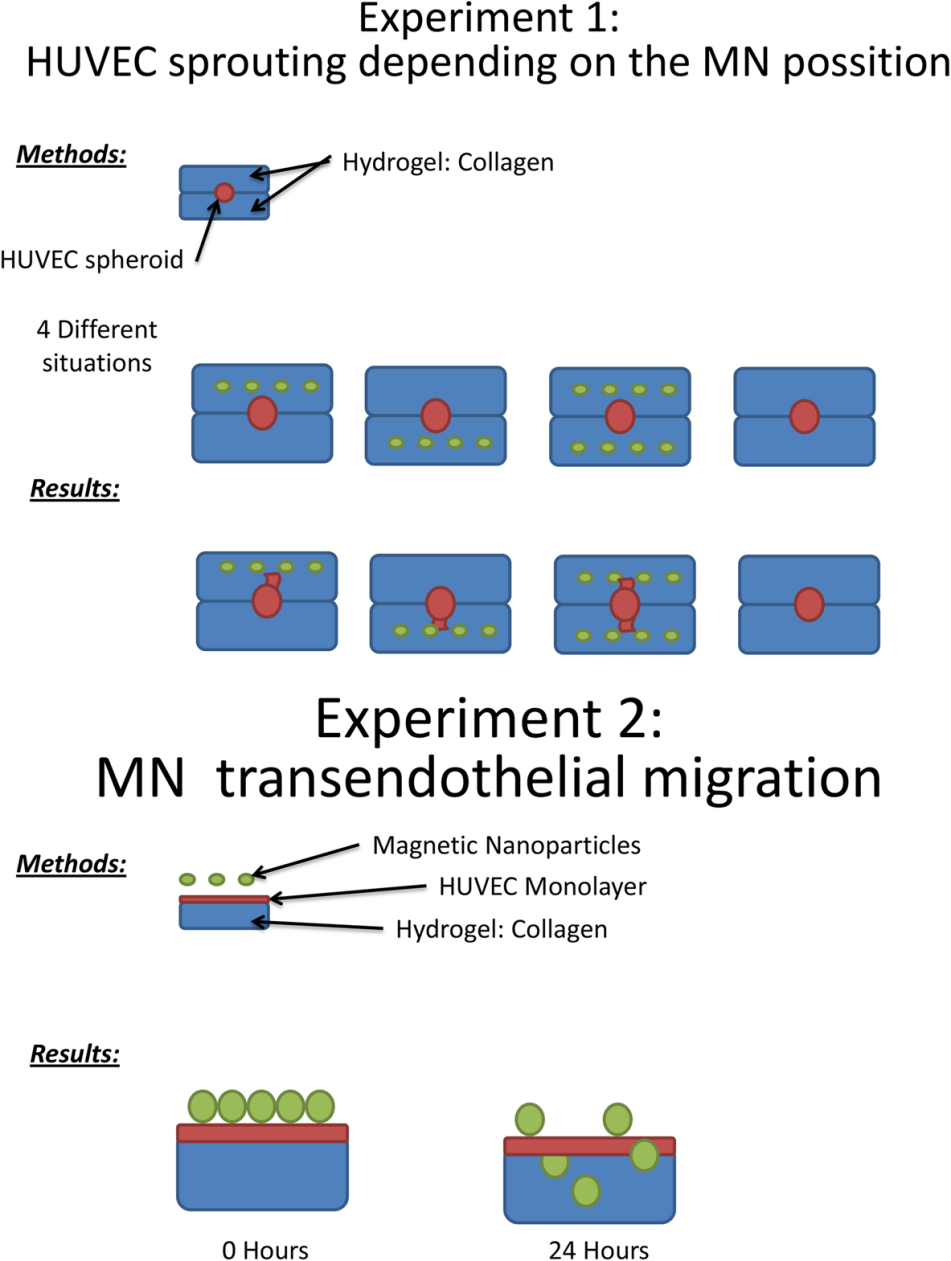
Schematic representation of the experiments conducted in the study – Experiments 1 and 2

HUVEC endothelial cells were modified to form clusters of HUVEC spheroids as the spheriods are better known to mimic natural cell responses and interactions [18, 19]. The extracellular matrix exerts its interaction with the cells, which again is influenced by the cellular architecture, and thereby determines the genetic and nuclear expression of the cells. This response is well observed with spheroids [20, 21]. This is especially useful for 3-D cell cultures due to its spheroidal shape. HUVECs are established to study angiogenesis or formation of capillary-like structures (CLS) in angiogenic engineering research [22, 23]. The tissue culture experiment was setup in four layers. The first layer had nutrients. The second layer was the collagen hydrogel layer; the third layer was a layer of HUVEC spheroids and the bottom layer was a layer of collagen hydrogel. The experiments were performed with nanoparticles in upper hydrogel layer, the lower hydrogel layer, and both layers and the results were compared to the control (Fig. 1).

**Fig. 2:**
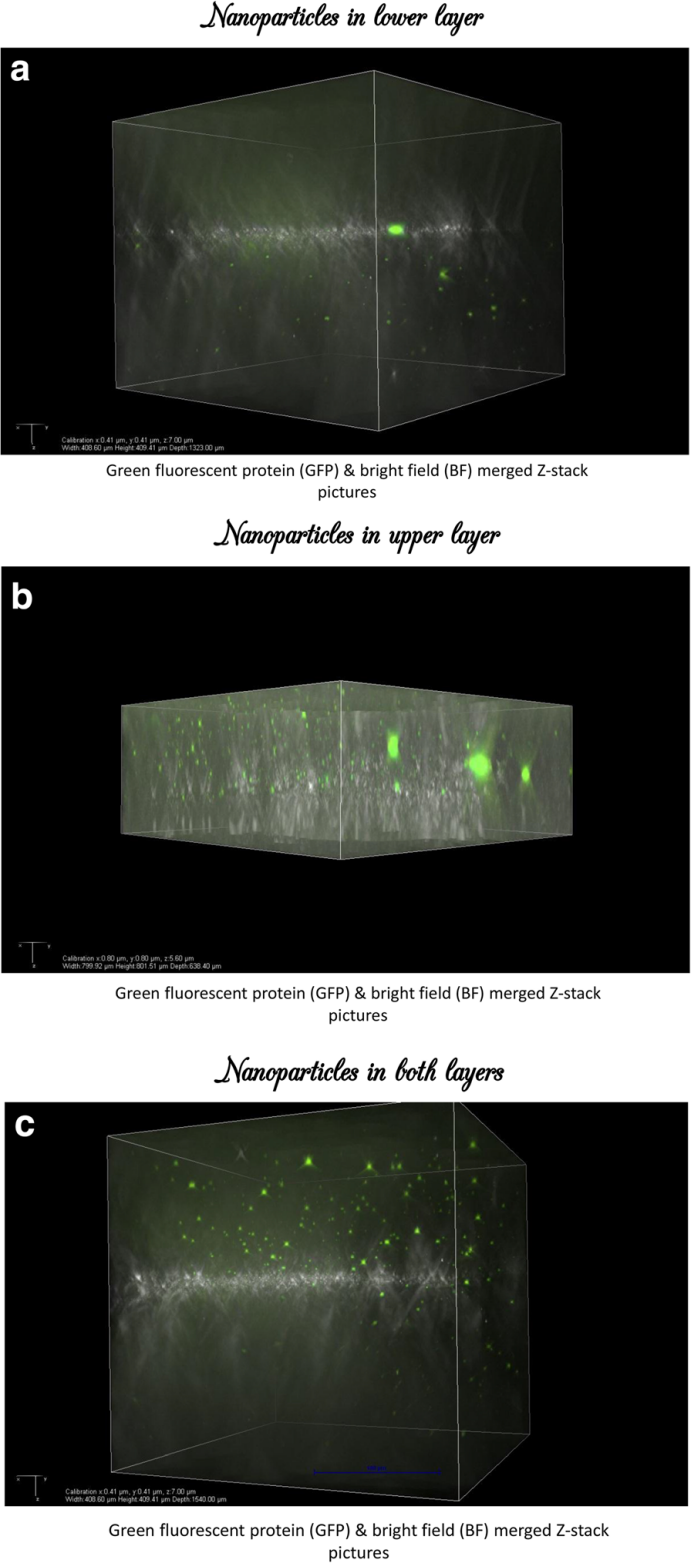
Green Fluorescent protein and Bright field merged Z-Stack pictures when nanoparticles were placed in lower (Panel **a**), upper (Panel **b**) and in both zones (Panel **c**)

The nanoparticles and the extent of the angiogenesis were studied using 2D-3Dimensional confocal microscopy with Z-stack fluorescent and bright field imaging. Serial pictures were acquired at regular intervals of 30 min were taken for 5 h in each experiment to study the extent of angiogenesis. Bright field imaging was used to study the extent of the spread of nanoparticles in hydrogel layer (Fig. 2). The nanoparticles in the hydrogel layer were manipulated using a bar magnet to desired locations for study. Two sets of experiment were performed in all the four cultures to study the extent and direction of angiogenesis. The concentration of the nanoparticles was the same in all the study experiments.

The nanoparticles could be used through intravascular or extravascular routes to form collaterals. Therefore, in experiment 2, which is the second phase of the study, the nanoparticles were studied for their effect on endothelial barrier crossing. The experiment was setup in a vertical sandwich structure inside a microfluidic chip with a monolayer of HUVEC cells, which was built on top of the collagen hydrogel layer. The nutrient layer with nanoparticles was placed above the HUVEC cell layer. The experiment was performed at 37 °C and 5% CO_2_. The ability and the extent of the nanoparticles to cross the HUVEC layer to reach the lower hydrogel layer were studied using Z-stack confocal microscopy. The confocal pictures were taken at 10 min and at 48 h to visualize the extent of crossing by the nanoparticles. The confocal observations were also made in serial intervals of time.

### Scanning electron microscopy

Scanning electron microscopy was performed with Ultra-Plus Zeiss microscope equipped with two secondary electron detectors: SE2 and In-Lens to characterize the size and distribution characteristics of the particles. The droplet was spread out on silicon at low speed rotation (700 rpm for 20s) and the sample was dried at 8000 rpm for 10s.

### Magnetic properties evaluation

The magnetic nanoparticles were evaluated by interfacing a fluid suspension (magnetic nanoparticles 1 mg/ml) with magnetic nanoparticles between a electromagnetic field. The experiment was repeated at varying magnetic filed strengths and the displacement of the nanoparticles were studied in four different times. The osmolarity of the droplet suspension was 320 mOsm/kg, which is near to serum osmolarity (280 mOsm/kg). In another experiment, a droplet of 4 mm size was placed over a bar-magnet (200 gauss) and its magnetic property was studied.

### Nuclear magnetic resonance imaging

NMR study was performed on the liquid sample with nanoparticles. A problem related to the paramagnetic nature of these particles was encountered. Indeed, interference with the magnetic field does not allow us to obtain an NMR signal, and a broad peak of water that covers the spectrum was seen. The signal of the nanoparticles was not visible due to the closeness of the super-paramagnetic iron oxide.

## Results

The scanning electron microscopy picture of the colloidal suspension of magnetic nanoparticles is shown in Additional file 1: Figure S1. NMR images obtained are shown in Additional file 1: Figure S2.

### Angiogenesis and microscopy

The angiogenesis sprouts were observed predominantly in the basal layer, and the direction of angiogenesis was from above downwards i.e. predominantly towards the basal layer. Additional file 2: Video S1 showed the effect of angiogenesis when nanoparticles were placed in the basal layer only. Additional file 3: Video S2 shows the effect of nanoparticles when placed in the upper layer of hydrogel. The sprouts were seen growing towards the upper nutrient layer. When the nanoparticles were placed in both the layers the sprouts were seen from bottom layer to the top (Additional file 4: Video S3). In the 4th scenario where there were no nanoparticles only, insignificant growth was seen (Additional file 5: Video S4). The results have proved that the nanoparticles with VEGF are efficient to grow the sprouts and the particles can be manipulated to desired locations in the hydrogel using a magnet.


Additional file 2: Video S1. The effects of magnetic nanoparticles with VEGF when placed in the basal layers. (WMV 466 kb)
Additional file 3: Video S2. The effects of magnetic nanoparticles with VEGF when placed in the upper layers. (WMV 1027 kb)
Additional file 4: Video S3. The effects of magnetic nanoparticles with VEGF when placed in both upper and lower basal layers. (WMV 991 kb)
Additional file 5: Video S4. Control experiment without nanoparticles. (WMV 435 kb)


### Endothelial barrier crossing

**Fig. 3:**
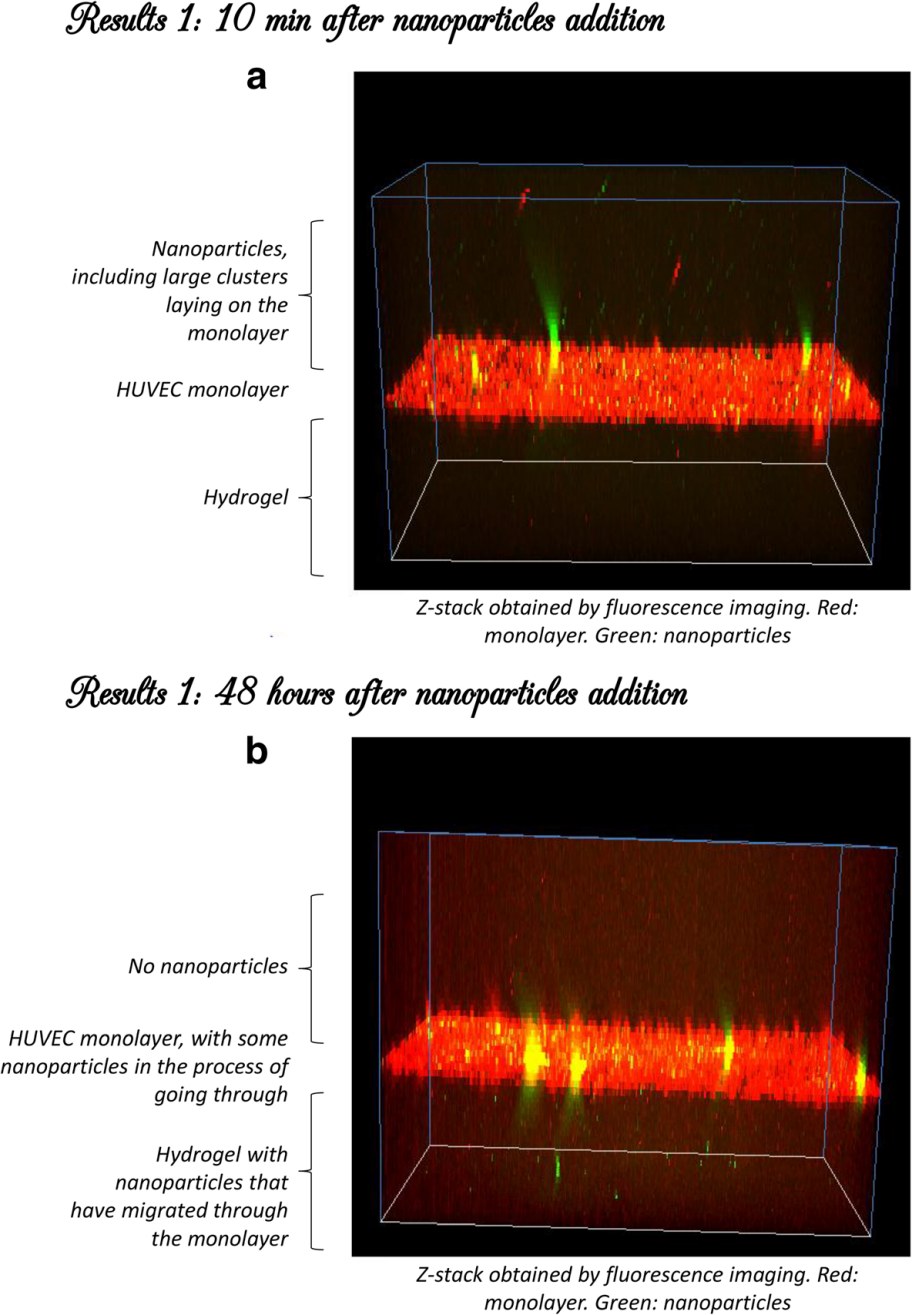
Panel **a** Z stack confocal microscopy pictures show the effect of endothelial barrier crossing of the nanoparticles at 10 min. Panel **b** show the effect of crossing the HUVEC monolayer at 48 h

In the next phase the endothelial barrier crossing of the nanoparticles was analyzed. Z-stack confocal microscopy was used to analyze the effect of the nanoparticles crossing the HUVEC monolayer to lower collagen hydrogel layer. It was noticed that at 10 min and 48 h the nanoparticles were crossing the endothelial barrier. The trans-endothelial migration was observed at 20 h. Quantification of the endothelial barrier crossing was not performed, however, by visual assessment a significant number of particles cross the barrier. Figure 3 a and b envisage the crossing of the nanoparticles (fluorescent particles) from the nutrient layer located above across the HUVEC monolayer cells to the lower collagen hydrogel layer located underneath.

### HUVEC-Nanoparticles interaction in Matrigel

**Fig. 4:**
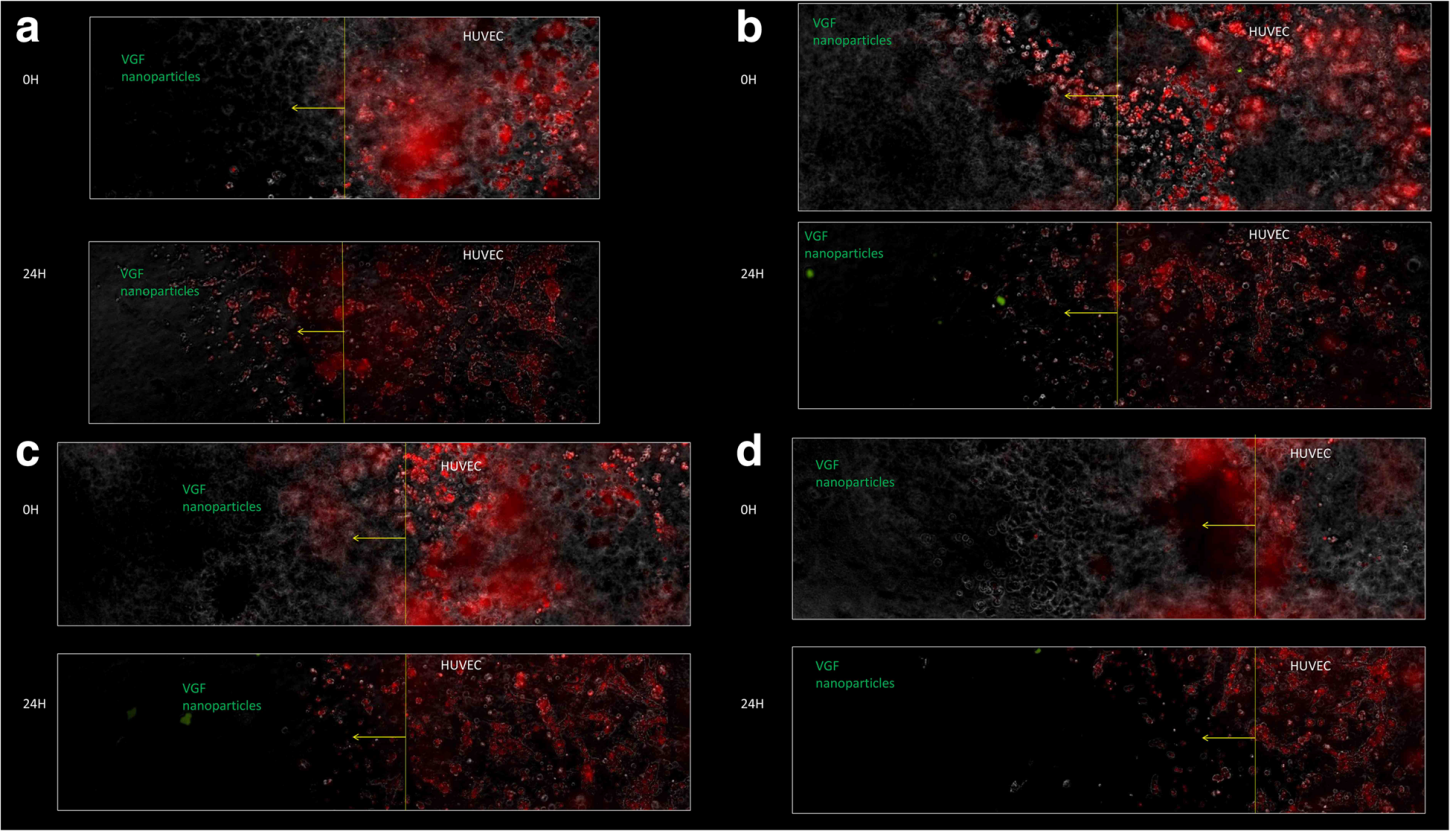
Shows disposition of the magnetic nanoparticles with VEGF and HUVECs in matrigel at 0 and 24 h. Panels **a** to **d** demonstrate the confocal images of 4 experiments in Matrigel observing the disposition of Magnetic Nanoparticles with VEGF on the left side of each panel and HUVEC cells on the right side. Panels **a** to **d**, each has upper and lower panels which correspond to immediate (0 hr), and at 24 hr after the experimental setup

The magnetic nanoparticles-VEGF particles were studied in matrix matrigel. The MN particles were placed on one side of the matrigel, and HUVECs were placed on the other side. The movement of the HUVECs was studied in standard matrigel. Figure 4 shows the disposition of the magnetic nanoparticles at 0 h and 24 h, and the demarcation line was set arbitrarily. There is a tendency for movement of the HUVECs towards magnetic nanoparticles conjugated with VEGF. Quantification of the HUVECs displacement was not performed, however, by visual assessment a significant number of HUVECs cross the demarcation line. In the presence of external magnetic force a significant displacement of MN + VEGF towards HUVECs is possible, which will facilitate angiogenesis.

### Magnetic polarization

**Fig. 5:**
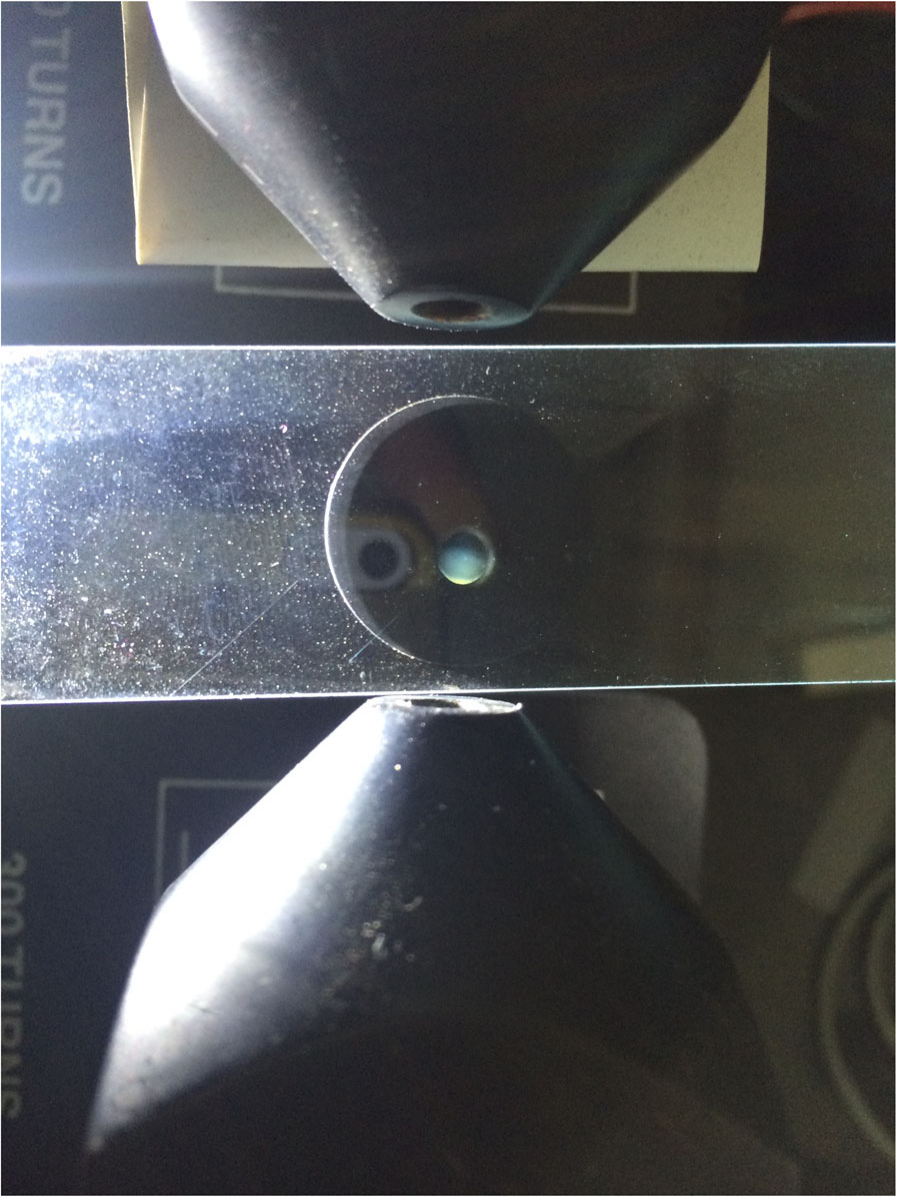
Shows polarization of the magnetic nanoparticles under the influence of 0.7 T electromagnetic field.

With electromagnetic interfacing the magnetic nanoparticles were observed to polarize to one end of the drop (Fig. 5). This was consistently observed in all 4 different times on a glass slide. The tendency of polarization starts at 0.1 T. Access to higher strengths of magnetic field was not available. Hence, the highest limit evaluated was 0.7 T.

In another experiment, the droplet with magnetic nanoparticles over a bar magnet retains its shape in all directions and antigravity positions (Additional file 1: Figure S3), whereas a control droplet without magnetic nanoparticles loses its shape very easily.

## Discussion

### Angiogenesis and collaterals

This study demonstrated the potential possibilities of angiogenesis using magnetic nanoparticles with VEGF. The results observed a proof-of-concept analysis of these nanoparticles showing the ability of the magnetic nanoparticles with VEGF to form angiogenic sprouts; and also the ability of MN to cross endothelial barrier. The advantage of the method is that the nanoparticles could be manipulated with magnetic force to desired locations, and angiogenesis thereby could be target oriented and therapeutic. This could be of particular use in the setting of coronary artery disease where the nanoparticles could be injected into the coronaries, and the particles could be potentially controlled by magnetic force to desired locations to form collaterals. The ability of these particles to move towards magnetic area, which was in the opposite direction to the nutrient layer, shows the influence of the magnetic field. Very interestingly it can be used through extravascular method to form arterial connections between left anterior descending artery and left internal mammary artery, which is placed adjacently and also the commonly used connection artery for coronary artery bypass surgery. The direction of the magnetic nanoparticle’s path could be controlled by targeted high field magnetic forces. To emphasize it has potentials to form percutaneous bypass connections, especially in patients who are not capable to undergo surgery due to comorbidities.

The potential advantages could be useful in treatment of peripheral vascular diseases and cerebral ischemic conditions. Also, this study has shown that these MN have potentials to cross the endothelial barrier, which could be of advantage. This was an experimental setup with a monolayer of HUVEC cells. However, in this setting, it is very difficult to simulate the exact tissue nature as the endothelium has a barrier and tight junctions. However, due to the nanostructure of the particles, it is expected to cross the endothelium easily, especially, if it is driven by an ischemic stimulus and with further magnetic field augmentation.

### Endothelial barrier crossing

It is difficult to simulate in-vitro the phenomenon of endothelial barrier crossing. A better method to demonstrate could have been usage of growth factor reduced matrigel, which could mimic tight junctions (Corning ™) instead of hydrogel used in this study [24, 25]. Matrix Matrigel is a recombinant basement membrane extract. Though absolute quantification of crossing was not studied this is the first evaluation of such a simulation. Moreover, this endothelial penetration property of the particles could be increased with external magnetic force.

### Magnetic manipulation

The magnetic particles show polarization in this study. This is the magnetic strength to overcome Vander Wall’s forces. The particles were evaluated in the past and the magnetic gradient required to control is 10 T/M [26].

The magnetic nanoparticles are small and they could be rapidly eliminated by monocytes. Aihua Fu et al., have generated high magnetic gradients using micro-mesh, and through these remarkable high gradients, the magnetic nanoparticles of even smaller sizes (<100 nm) could be controlled to retain them in intravascular compartment [26]. The particles accumulated in the target areas using the Ni micro-mesh technique. The Ni mesh used in the study was 76 μm pitch, 12 μm wire width and 5 μm wire thickness, and using this large gradients of 10^5^ to 10^10^ gradient were developed within 10 μm of Nitinol edges.

The magnetic nanoparticles could also be injected extravasularly to form collateral connections between left anterior descending artery and left internal mammary or intercostal vessels (percutaneous bypass); and thereafter-magnetic particles can be externally controlled by the external magnetic field.

### Future potential applications

These particles could be injected through percutaneous or sub-sternal/subcostal route, and an attempt to form collaterals from internal mammary to the coronaries i.e. in the form of a percutaneous bypass technique, as a drive for future research would be very interesting. Targeted nanoparticles are useful to identify tumor cells, modulation of sRNA and cardiosphere derived cells engraftment in myocardial infarction [27–29]. The magnetic nanoparticles are useful for receptor-mediated gene delivery [30]. Also, suppression of VEGF has been shown to be associated with regression of the tumor, and also in the control siRNA, and receptor-mediated signal transduction [31]. Also, it would be quite interesting to study the effect of integrin expression on the cardiomyocyte membranes by these nanoparticles, as VEGF expression can induce integrin expression in cardiomyocyte surface also, which could emerge as a dual advantage [32, 33]. These particles need to be studied in-vitro for further information on the magnetic displacement of the particles.

### Biocompatibility and degradation

These MN are largely biocompatible, and it could be used for various bioengineering purposes in vitro and in vivo [34]. Magnetic levitation is a technique of modification of the 3D cell culture with magnetic nanoparticles and studying the modified effect of the tissues used in culture [35]. The cellular behavior could be controlled in a remote control form [36]. Magnetic nanoparticles are very useful in bioengineering of the tissues and antibody purification. The magnetic nanoparticles are useful in-vivo gene delivery, antibody purification and photo-acoustic detection of circulating tumor cells.

### Limitations

This study is an initial observation demonstrating the ability of magnetic nanoparticles with VEGF in angiogenesis and endothelial barrier crossing. Further studies need to be preformed to look for the extent, and the magnitude of targeted angiogenesis.

## Conclusion

Angiogenesis could be induced by the effect of magnetic nanoparticles conjugated with the vascular endothelial growth factor. These nanoparticles could be controlled by magnetic force, and these nanoparticles have possible potentials to cross endothelium.

## Additional files

Additional file 1: Figure S1. Shows Scanning electron microscope images of magnetic nanoparticles. **Figure S2.** Nuclear magnetic resonance imaging of the particle suspension. **Figure S3.** shows pictures of droplet with magnetic nanoparticles in different positions of the magnet. (DOCX 1634 kb)

## Abbreviations

HUVEC: Human umbilical vascular endothelial cells
MN: Magnetic nanoparticles
VEGF: Vascular endothelial growth factor
