# Supplementary material for: A novel targeted angiogenesis technique using VEGF conjugated magnetic nanoparticles and in-vitro endothelial barrier crossing
Source: BMC Cardiovasc Disord. 2017 Jul 28;17:209. doi: 10.1186/s12872-017-0643-x (PMC5534071; doi:10.1186/s12872-017-0643-x)

**Additional file 1**

**A Novel Targeted Angiogenesis Technique Using VEGF Conjugated Magnetic Nanoparticles And *In-Vitro* Endothelial Barrier Crossing**

**Mark C. Arokiaraj***

Supplemental FIGURE 1 shows Scanning electron microscope images of magnetic nanoparticles


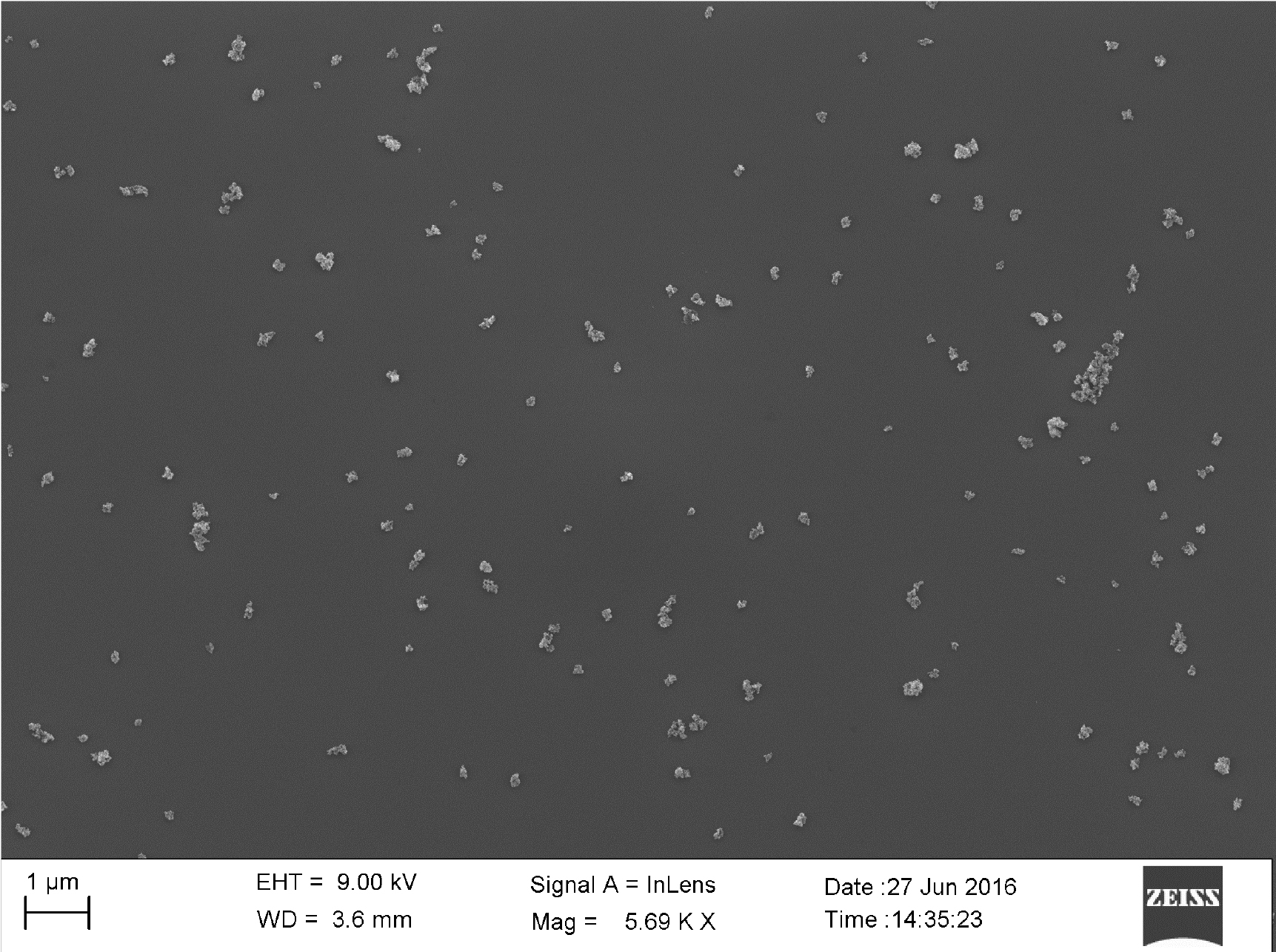


Supplemental FIGURE 1 shows scanning electron microscope images of magnetic nanoparticles

Supplementary Figure 3 Nuclear magnetic resonance imaging of the particle suspension

**
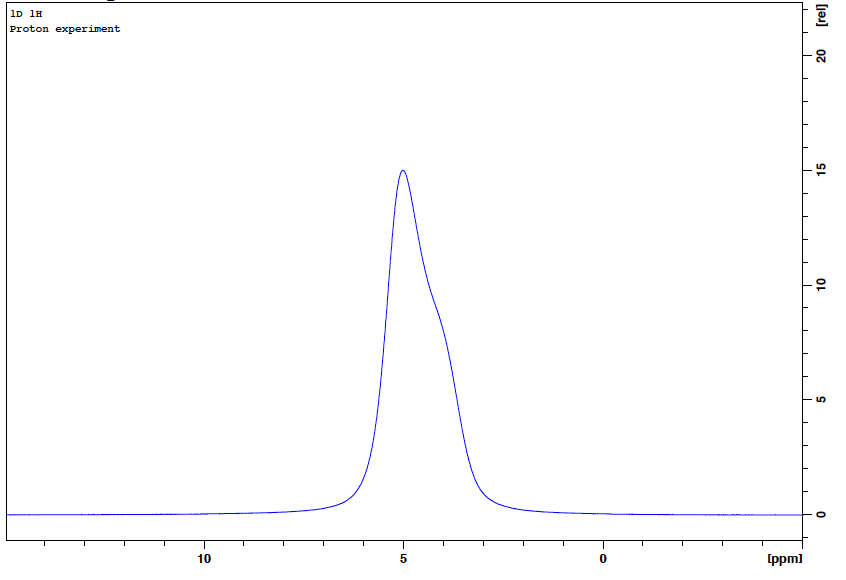
**


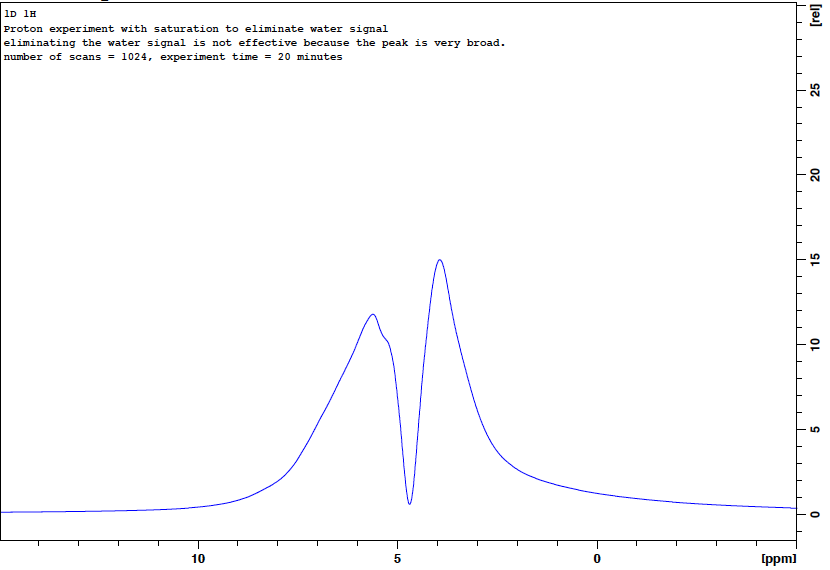


Supplemental FIGURE 3 shows pictures of droplet with magnetic nanoparticles in different positions of the magnet.


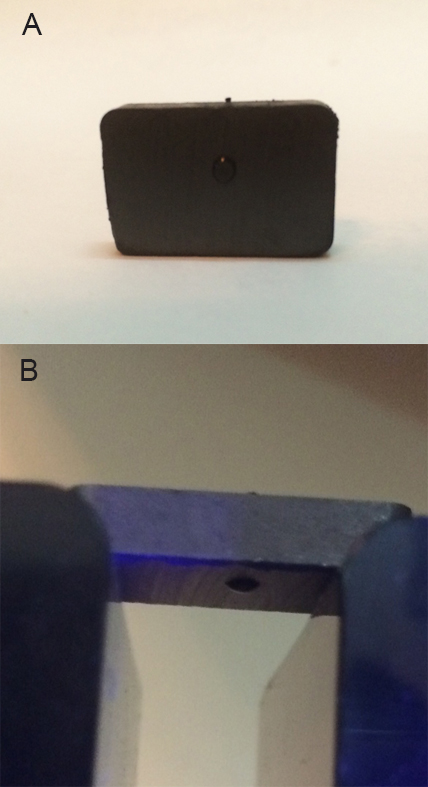

Supplement: Supplementary file 5 — Shows Scanning electron microscope images of magnetic nanoparticles. Figure S2. Nuclear magnetic resonance imaging of the particle suspension. Figure S3. shows pictures of droplet with magnetic nanoparticles in different positions of the magnet. (DOCX 1634 kb) [file 12872_2017_643_MOESM1_ESM.docx]
